# Detoxified pneumolysin derivative ΔA146Ply inhibits autophagy and induces apoptosis in acute myeloid leukemia cells by activating mTOR signaling

**DOI:** 10.1038/s12276-022-00771-7

**Published:** 2022-05-10

**Authors:** Tao Zhu, Hong Zhang, Sijie Li, Kaifeng Wu, Yibing Yin, Xuemei Zhang

**Affiliations:** 1 Department of Laboratory Medicine, Key Laboratory of Diagnostic Medicine (Ministry of Education), Chongqing Medical University, Chongqing, 400016 China; 2 Department of Clinical Laboratory, Chongqing Key Laboratory of Translational Research for Cancer Metastasis and Individualized Treatment, Chongqing University Cancer Hospital & Chongqing Cancer Institute & Chongqing Cancer Hospital, Chongqing, 400030 China; 3 Department of Laboratory Medicine, The Affiliated Hospital of North Sichuan Medical College, and Department of Laboratory Medicine and Translational Medicine Research Center, North Sichuan Medical College, Nanchong, 637000 China; 4 Department of Laboratory Medicine, the Third Affiliated Hospital of Zunyi Medical University, Zunyi, 563000 China

**Keywords:** Translational research, Acute myeloid leukaemia

## Abstract

Leukemia is caused by the malignant clonal expansion of hematopoietic stem cells, and in adults, the most common type of leukemia is acute myeloid leukemia (AML). Autophagy inhibitors are often used in preclinical and clinical models in leukemia therapy. However, clinically available autophagy inhibitors and their efficacy are very limited. More effective and safer autophagy inhibitors are urgently needed for leukemia therapy. In a previous study, we showed that ΔA146Ply, a mutant of pneumolysin that lacks hemolytic activity, inhibited autophagy of triple-negative breast cancer cells by activating mannose receptor (MR) and toll-like receptor 4 (TLR4) and that tumor-bearing mice tolerated ΔA146Ply well. Whether this agent affects AML cells expressing TLR4 and MR and the related mechanisms remain to be determined. In this study, we found that ΔA146Ply inhibited autophagy and induced apoptosis in AML cells. A mechanistic study showed that ΔA146Ply inhibited autophagy by activating mammalian target of rapamycin signaling and induced apoptosis by inhibiting autophagy. ΔA146Ply also inhibited autophagy and induced apoptosis in a mouse model of AML. Furthermore, the combination of ΔA146Ply and chloroquine synergistically inhibited autophagy and induced apoptosis in vitro and in vivo. Overall, this study provides an alternative effective autophagy inhibitor that may be used for leukemia therapy.

## Leukemia: A new combination to kill cancer cells

A mutated form of the bacterial protein pneumolysin offers a new approach to treating acute myeloid leukemia (AML), due to its ability to stimulate cancer cells to undergo a form of cell suicide called apoptosis. Researchers in China led by Xuemei Zhang at Chongquing Medical University studied the effects of a pneumolysin derivative on cultured human and mouse AML cells. They identified the mechanism by which this derivative activates a known molecular signaling system to inhibit the process of autophagy, in which cells routinely ‘clean up’ degraded or unnecessary components during normal maintenance. This inhibition of autophagy then induced the apoptosis that killed cancer cells. The effect became more pronounced when the pneumolysin derivative was combined with the existing autophagy-inhibiting drug chloroquine. The new combination could be safer and more effective than using chloroquine alone.

## Introduction

Leukemia is caused by the malignant clonal expansion of hematopoietic stem cells and is the second leading cause of cancer deaths in men under the age of 40 and in women under the age of 20 in the United States^1,2^. In adults, the most common type of leukemia is acute myeloid leukemia (AML)^3^. Clinically, chemotherapy remains the main treatment for AML. However, leukemia cells have developed multiple mechanisms to resist chemotherapeutic agents, which leads to the failure of AML therapy, making prognosis unsatisfactory. More than half of patients will ultimately die from this disease^4–6^.

Autophagy plays complicated roles in the development and treatment of cancer^7,8^. A well-accepted paradigm in leukemia is that autophagic dysfunction contributes to leukemogenesis, while in leukemia therapy, autophagy activation promotes the survival of leukemia cells by removing excess metabolites and damaged mitochondria^9–12^. Only in some circumstances does autophagy activation benefit leukemia treatment by degrading certain oncoproteins^13–15^. Therefore, autophagy inhibitors are often used in preclinical and clinical models for leukemia therapy^16–18^. Currently, chloroquine (CQ) is the only clinically available autophagy inhibitor for use in humans. Its efficacy is positively correlated with its dose. To achieve better efficacy, high doses of CQ are needed. However, when used at high doses, CQ may cause serious side effects, including electrocardiographic changes, visual disturbances, and gastrointestinal reactions^19–21^. More effective and safer autophagy inhibitors are urgently needed for leukemia therapy.

ΔA146Ply, a mutant of pneumolysin (Ply) that lacks hemolytic activity, has been shown to inhibit autophagy in triple-negative breast cancer cells by activating toll-like receptor 4 (TLR4) and mannose receptor (MR), which suggests that ΔA146Ply may act on other cells that express TLR4 and MR. It has been well established that TLR4 and MR are highly expressed on the surface of myeloid cells^22^. Therefore, we hypothesize that ΔA146Ply acts on AML cells. However, the effect of ΔA146Ply on AML cells remains to be determined.

In the present study, the human AML cell line KG-1 and the mouse AML cell line C1498 were used, and we demonstrated that ΔA146Ply inhibited autophagy and induced apoptosis in AML cells. A mechanistic study showed that ΔA146Ply inhibited autophagy by activating mammalian target of rapamycin (mTOR) signaling and induced apoptosis by inhibiting autophagy. ΔA146Ply also inhibited autophagy and induced apoptosis in a mouse model of AML. Furthermore, the combination of ΔA146Ply and CQ synergistically inhibited autophagy and induced apoptosis both in vitro and in vivo. In summary, this study provides an alternative effective autophagy inhibitor that may be used for leukemia therapy.

## Materials and methods

### Reagents

Polymyxin B agarose and Ni^2+^-charged column chromatographs were provided by Genscript (New Jersey, USA) and GE Healthcare (Buckinghamshire, UK), respectively. Mouse monoclonal antibodies, including anti-glyceraldehyde 3-phosphate dehydrogenase (GAPDH), anti-B-cell lymphoma 2 (BCL-2), and anti-Bax, were provided by Santa Cruz Biotechnology Corporation (Santa Cruz, CA). Rabbit monoclonal antibodies, including anti-LC3A/B, anti-p62, anti-CC3, anti-phospho-Akt, anti-Akt, anti-phospho-ERK, anti-ERK, and anti-phospho-mTOR, were provided by Cell Signaling Technology Corporation (Beverly, MA). The TNF-α ELISA kit, mouse PE-labeled TLR4 antibodies, mouse APC-labeled MR antibodies, human PE-labeled antibodies antibody, and human APC-labeled MR antibodies were purchased from Biolegend Corporation. MK2206 and U0126 were purchased from Selleck Corporation and dissolved in dimethylsulfoxide (DMSO). CQ and BBM were provided by Sigma–Aldrich Corporation (St Louis, MO, USA).

### Cell lines

The mouse AML cell lines C1498 and Raw264.7, human chronic myelogenous leukemia cell line K562, and human AML cell lines KG-1 and THP-1 were provided by the American Type Culture Collection (ATCC). RPMI-1640 medium (Gibco) supplemented with 1% penicillin–streptomycin (HyClone, USA) and 10% fetal bovine serum (FBS) (Biological Industries, Israel) was used to culture these cells at 37 °C in 5% CO_2_.

### Mice

Specific pathogen-free, 5- to 6-week-old female C57BL/6J mice were provided by Beijing SPF Biotechnology Co., Ltd. (Beijing, China). The mice were maintained in barrier conditions with mouse chow and sterile water ad libitum at Chongqing Medical University. The Ethics Committee of Chongqing Medical University approved all procedures for animal experiments.

### Preparation of ΔA146Ply and PepO

In this study, the pneumococcal virulence protein PepO was used as a protein control^23^. ΔA146Ply and PepO were prepared as previously described^23,24^. Protein purification was performed with a Ni^2+^-charged column chromatograph. Polymyxin B agarose was used to remove lipopolysaccharide from the protein preparations. The protein concentration was determined by ultraviolet spectrophotometry.

### Flow cytometry analysis

For cell surface receptor analysis, cells in six-well plates were collected, washed twice with prechilled PBS, blocked with 50 μl of rat serum for 20 min, and incubated with PE-labeled TLR4 and APC-labeled MR antibodies or corresponding isotype antibodies in the dark for 30 min. Then, the cells were washed and resuspended in PBS for analysis. Fluorescein isothiocyanate-labeled Annexin V and phosphatidylinositol (PI) were used to stain the treated cells to analyze apoptosis. For cell cycle analysis, prechilled 75% ethanol was used to fix the cells, and PI was used to stain the cells. All samples were analyzed with a BD LSRFortessa cell analyzer.

### Western blot analysis

KG-1 or C1498 cells were treated accordingly. Before being collected, the cells were washed twice with prechilled PBS. Then, the collected cells were lysed with radioimmunoprecipitation assay (RIPA; BiYunTian, Shanghai, China) containing protease and phosphorylase inhibitors (Roche) and sodium dodecyl sulfate (SDS) loading buffer. After being boiled for 10 min, the samples were centrifuged at 13,000 rpm for 10 min to remove cell debris. To separate the proteins, SDS–PAGE was performed, and the separated proteins were transferred to a polyvinylidene fluoride membrane. Then, the membrane was blocked with 5% nonfat milk, incubated with the indicated primary antibodies, washed four times, incubated with the corresponding secondary antibodies, and washed five times. Then, a Bio–Rad chemiluminescence detection system was used to detect the specific protein bands, and GAPDH expression was used as an endogenous reference.

### Immunofluorescence analysis

KG-1 or C1498 cells were treated with ΔA146Ply (10 μg/ml) for 6 h. The cells were washed with prechilled PBS three times and fixed on glass coverslips for immunofluorescence staining. Then, the cells were fixed with 4% paraformaldehyde, permeabilized with 0.1% Triton X-100, blocked with 5% bovine serum albumin, and incubated with anti-LC3A/B antibodies (1:200 dilution). Following three washes, the cells were incubated with Alexa Fluor 488-conjugated secondary antibodies and stained with 4′,6-diamidino-2-phenylindole. A Nikon ECLIPSE 80i microscope was used to observe LC3 puncta.

### Enzyme-linked immunosorbent assay (ELISA)

A specific ELISA kit (Biolegend) was used to measure TNF-α levels in the culture supernatant of KG-1 and C1498 cells, and the experiment was performed according to the manufacturer’s instructions.

### In vivo experiments

The mouse AML model was established by intravenous injection of 5 × 10^6^ C1498 cells into C57BL/6J mice. Twelve days after C1498 administration, the mice were intraperitoneally injected with CQ (50 mg/kg) and ΔA146Ply (200 μg per mouse) individually or in combination. CQ was injected every day, and ΔA146Ply was injected every 4 days. After four rounds of ΔA146Ply administration, the mice were euthanized, and blood samples, femurs, livers, spleens, and lung tissues were collected.

### Immunohistochemistry

After being dewaxed with xylene and hydrated with an ethanol series, the bone marrow sections and smears were incubated in sodium citrate at 95 °C for epitope retrieval, treated with 3% H_2_O_2_ to inhibit endogenous peroxidase, blocked with goat serum, and stained with monoclonal rabbit anti-LC3, anti-p62, and anti-CC3 antibodies. After being incubated with biotin-labeled secondary antibodies and streptavidin-horseradish peroxidase, the tissues were developed with 3,3′-diaminobenzidine, followed by hematoxylin counterstaining. Then, the samples were observed under a light microscope.

### Hematoxylin and Eosin (HE) staining

After being fixed with 4% paraformaldehyde, dehydrated with an ethanol series, and embedded in paraffin, the collected liver, spleen, and lung tissues were sectioned into 5 μm serial sections. Before being observed under a light microscope, the sections were stained with hematoxylin-eosin.

### Blood cell and biochemical analysis

An automatic blood cell analyzer (Sysmex, Japan) was used to analyze blood cells, and an e702 automatic biochemical analyzer (Roche, Germany) was used to measure serum ALT, AST, and urea levels. The samples were analyzed according to the manufacturer’s instructions.

### Cell Counting Kit 8 (CCK8) assay

Leukemia cells were treated accordingly at 37 °C for the indicated times. CCK8 reagent (Bimake) was used to determine cell viability, and this process was performed according to the manufacturer’s instructions. The percent viability was calculated by normalizing the absorbance values at 450 nm of the treated group to that of the control group.

### Transwell assay

Boyden chambers with filters (8 µm pore size, Corning Costar) were used to perform cell migration and invasion assays. For the invasion assay, the chamber was coated with 100 µl of Matrigel (BD) before use. Briefly, RPMI 1640 medium containing 20% FBS was added to the lower chamber, and KG-1 cells were seeded in the upper chamber. After treatment, 4% paraformaldehyde was used to fix cells on the filters, and 0.1% crystal violet in PBS was used to stain the cells. Cells on the upper side of the filters were removed with swabs, and the migrating or invading cells were observed under a phase-contrast microscope. The cells in the lower chamber were collected and counted with a Neubauer hemocytometer.

### Statistical analysis

GraphPad Prism 5 statistical software (La Jolla, CA, USA) was used to analyze all data. The data are shown as the mean ± standard deviation (SD). Student’s *t* test was used to determine the difference between groups. For all analyses, differences with *p* < 0.05 were considered significant.

## Results

### ΔA146Ply inhibits autophagy in KG-1 and C1498 cells

**Fig. 1:**
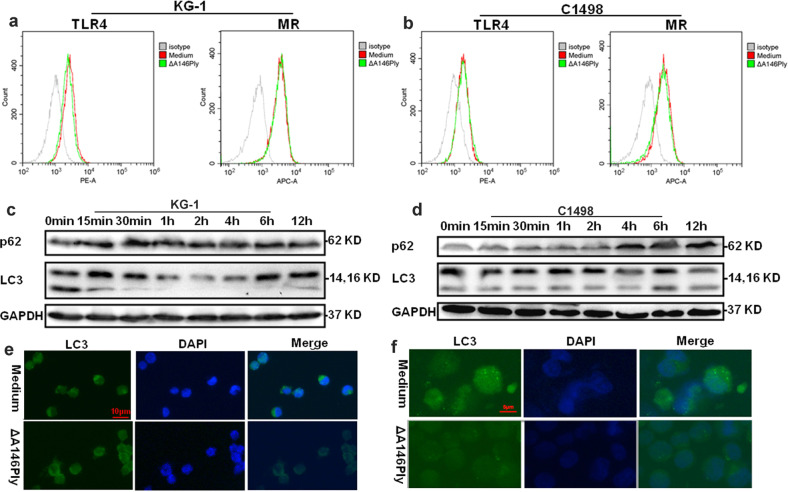
ΔA146Ply inhibits autophagy in KG-1 and C1498 cells. Surface expression of TLR4 and MR on KG-1 **a** and C1498 **b** cells was analyzed by flow cytometry. Intracellular autophagy-associated proteins in KG-1 **c** and C1498 **d** cells treated with ΔA146Ply (10 μg/ml) for different times were measured by western blotting. Intracellular LC3 puncta in KG-1 **e** and C1498 **f** cells treated with ΔA146Ply for 6 h were measured by immunofluorescence analysis.

Our previous study proved that ΔA146Ply inhibited autophagy in triple-negative breast cancer cells by activating TLR4 and MR. To determine whether ΔA146Ply affected AML cells, flow cytometry was performed to analyze TLR4 and MR expression on the surface of AML cells. The data in Fig. 1a, b showed that both TLR4 and MR were expressed on KG-1 and C1498 cells. To further determine whether ΔA146Ply can inhibit autophagy in KG-1 and C1498 cells, autophagy-associated proteins in ΔA146Ply-treated KG-1 and C1498 cells were measured by western blotting and immunofluorescence staining. As shown in Fig. 1c, d, after ΔA146Ply treatment, p62 levels in KG-1 and C1498 cells increased, and LC3-II levels in KG-1 cells decreased. Furthermore, the number of LC3 puncta in ΔA146Ply-treated KG-1 and C1498 cells decreased (Fig. 1e, f). These results proved that ΔA146Ply inhibited autophagy in KG-1 and C1498 cells.

### ΔA146Ply activates mTOR signaling

**Fig. 2:**
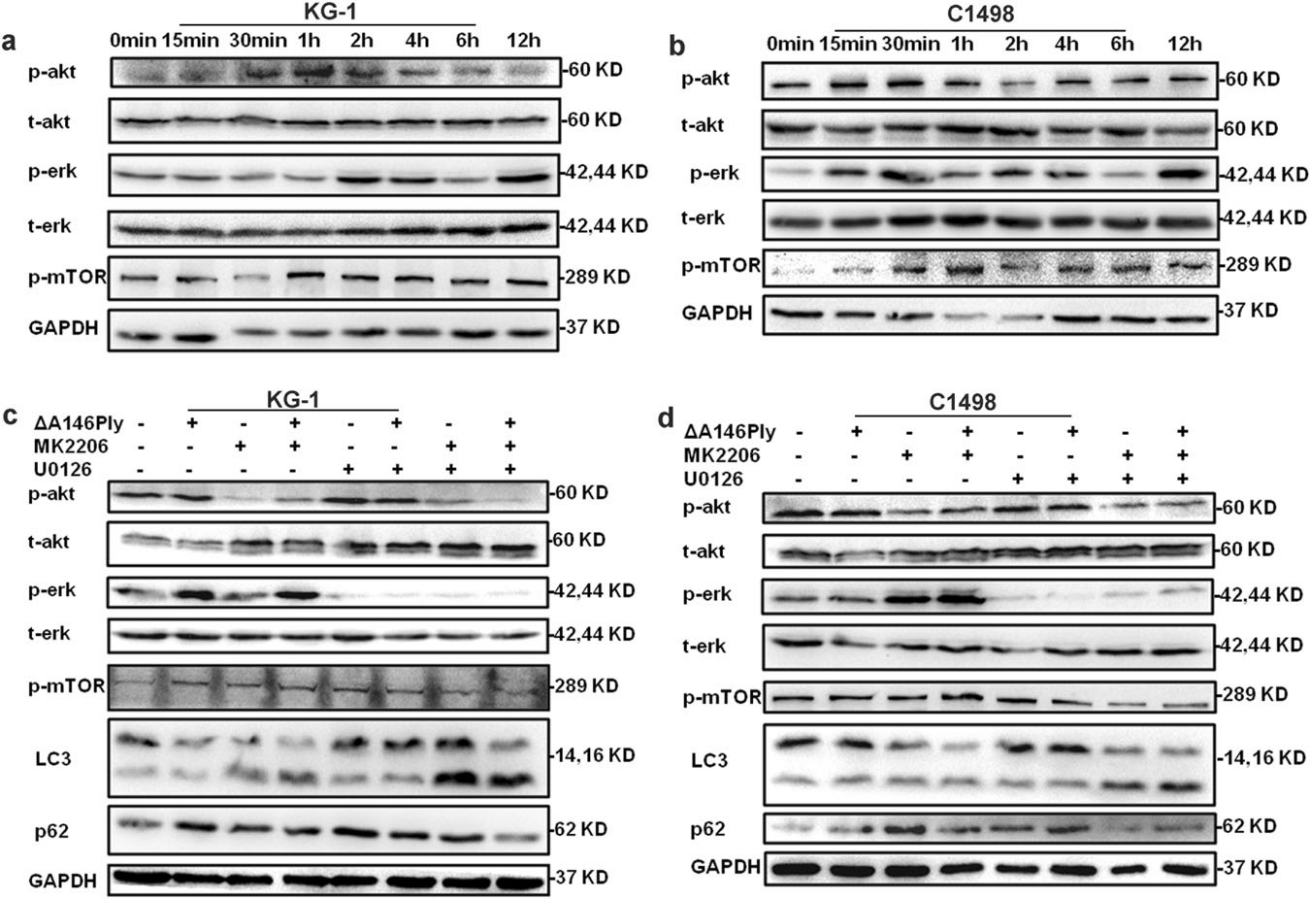
ΔA146Ply inhibits autophagy by activating mTOR signaling. Intracellular proteins in KG-1 **a** and C1498 **b** cells treated with ΔA146Ply (10 μg/ml) for different times were measured by western blotting. After 1 h of pretreatment with MK2206 (2.5 μM) or U0126 (20 μM), KG-1 **c** and C1498 **d** cells were incubated with ΔA146Ply for an additional 4 h. Intracellular proteins were measured by western blotting.

Our previous study proved that ΔA146Ply inhibited autophagy in triple-negative breast cancer cells by activating mTOR signaling. To determine whether mTOR signaling was responsible for ΔA146Ply-induced autophagy inhibition in KG-1 and C1498 cells, western blotting was performed. The data in Fig. 2a, b show that in ΔA146Ply-treated KG-1 and C1498 cells, the phosphorylation levels of Akt, extracellular signal-regulated kinase (ERK), and mTOR increased, and peak phosphorylation of mTOR occurred at 1 h. To further determine whether autophagy inhibition is mediated by the activation of mTOR signaling, we targeted factors upstream of mTOR signaling because of the nonspecific toxic effect of mTOR inhibitors, including rapamycin and PP242, on leukemic cells^25–28^. It is well established that both Akt and ERK are upstream signaling molecules of mTOR, and we measured their phosphorylation levels in ΔA146Ply-treated KG-1 and C1498 cells^29–32^. Therefore, MK2206, an Akt small molecular inhibitor, and U0126, an ERK small molecular inhibitor, were used in our experiments. After being pretreated with MK2206 or U0126 for 1 h, the cells were stimulated with ΔA146Ply for an additional 4 h. Western blotting was performed to measure intracellular autophagy-associated proteins. The data in Fig. 2c, d show the effective inhibition of Akt and ERK phosphorylation mediated by MK2206 and U0126. After MK2206 and U0126 pretreatment, ΔA146Ply failed to phosphorylate mTOR and increase p62 levels in KG-1 and C1498 cells. These results indicated that ΔA146Ply inhibited autophagy in KG-1 and C1498 cells by activating mTOR signaling.

### ΔA146Ply induces apoptosis in KG-1 and C1498 cells

**Fig. 3:**
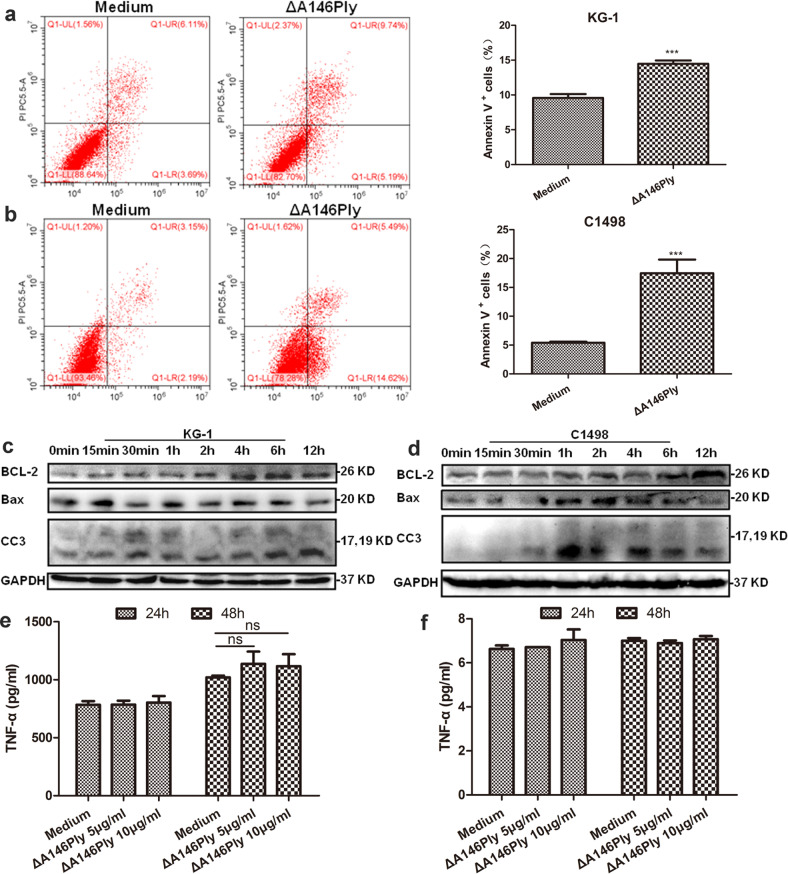
ΔA146Ply induces apoptosis in KG-1 and C1498 cells. After treatment with ΔA146Ply (10 μg/ml) for 48 h, apoptotic KG-1 **a** and C1498 **b** cells were analyzed by flow cytometry. Representative pictures are shown. The mean percentage ± SD of Annexin V^+^ cells is shown in the graphs (*n* = 3). Intracellular proteins in KG-1 **c** and C1498 **d** cells treated with ΔA146Ply (10 μg/ml) for different times were measured by western blotting ELISA was performed to measure TNF-α levels in the culture supernatant of KG-1 **e** and C1498 **f** cells treated with different concentrations of ΔA146Ply for 24 h or 48 h. Student’s *t* test was used to analyze significant differences. ***p < 0.001, ns no significance.

Increasing evidence has demonstrated that autophagy inhibition contributes to apoptosis induction^33–35^, especially in tumor cells. To determine whether ΔA146Ply can induce apoptosis in KG-1 and C1498 cells, the cells were treated with ΔA146Ply, and apoptotic cells were analyzed by flow cytometry. The data in Fig. 3a, b show that ΔA146Ply induced apoptosis in KG-1 and C1498 cells.

It is well established that two apoptosis pathways exist in cells: the intrinsic and extrinsic pathways^36,37^. The antiapoptotic protein BCL-2 and proapoptotic protein BCL-2-associated X protein (Bax) are important regulators of the intrinsic apoptosis pathway, and the binding of death ligands, including TNF-α, to death receptors initiates the extrinsic apoptosis pathway. Therefore, we evaluated the effect of ΔA146Ply on these apoptosis pathways by measuring BCL-2, Bax, and TNF-α expression. As shown in Fig. 3c, d, after ΔA146Ply treatment, cleaved caspase 3 (CC3) levels in KG-1 and C1498 cells increased, and an initial increase occurred at 30 min. In addition, we found that BCL-2 expression increased and Bax expression decreased, especially after 12 h, indicating that the intrinsic apoptosis pathway may not be initiated by ΔA146Ply treatment. TNF-α levels in the culture supernatant of KG-1 and C1498 cells in the ΔA146Ply group did not increase compared with those in the medium group (Fig. 3e, f), suggesting that the extrinsic apoptosis pathway may not be initiated by ΔA146Ply treatment. Taken together, these results indicated that apoptosis induction by ΔA146Ply in KG-1 and C1498 cells may be mediated by autophagy inhibition.

### ΔA146Ply induces apoptosis by inhibiting autophagy

**Fig. 4:**
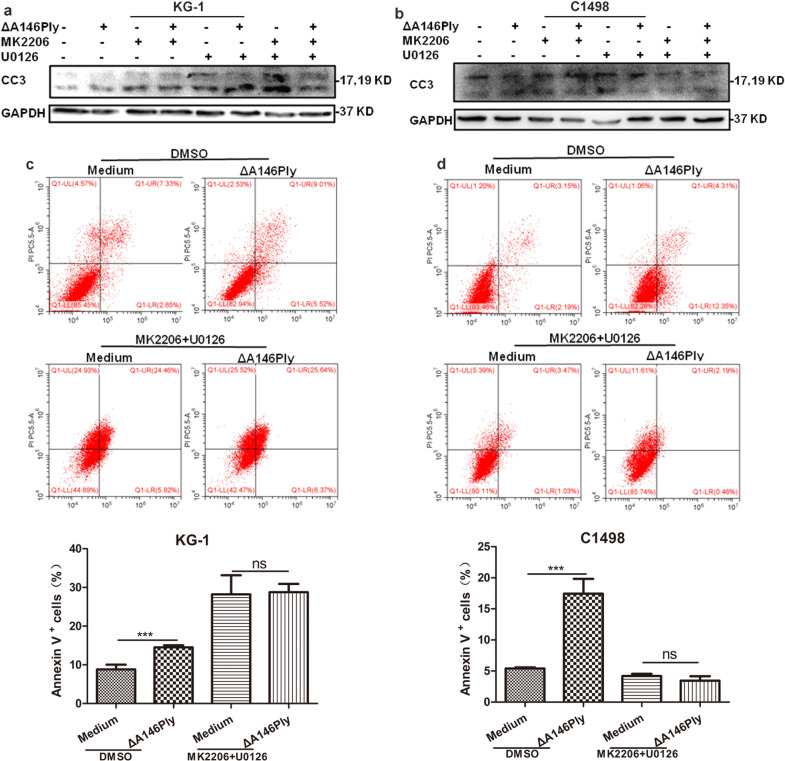
ΔA146Ply induces apoptosis by inhibiting autophagy. After 1 h of pretreatment with MK2206 (2.5 μM) or U0126 (20 μM), KG-1 **a** and C1498 **b** cells were treated with ΔA146Ply for an additional 4 h, and western blotting was performed to measure intracellular proteins. After 1 h of pretreatment with MK2206 (2.5 μM) or U0126 (20 μM), KG-1 **c** and C1498 **d** cells were treated with ΔA146Ply for an additional 4 h, and flow cytometry was used to analyze apoptotic cells. Representative pictures are shown. The mean percentage ± SD of Annexin V^+^ cells is shown in the graphs (*n* = 3). Student’s *t* test was used to analyze significant differences. ***p < 0.001, ns no significance.

To determine whether apoptosis induction by ΔA146Ply in KG-1 and C1498 cells was a result of autophagy inhibition, the effect of autophagy inhibition was blocked by MK2206 and U0126 pretreatment, and the effect of ΔA146Ply on apoptosis was analyzed by western blotting and flow cytometry. As shown in Fig. 4a, b, after MK2206 and U0126 pretreatment, ΔA146Ply failed to increase CC3 levels in either KG-1 or C1498 cells. Furthermore, ΔA146Ply also failed to induce apoptosis in KG-1 and C1498 cells in the presence of MK2206 and U0126 (Fig. 4c, d). Unexpectedly, we found that MK2206 and U0126 treatment increased apoptosis in KG-1 cells but not C1498 cells. The data in Fig. 2c, d show that MK2206 and U0126 treatment inhibited autophagy in KG-1 but not C1498 cells, which may account for this phenomenon. Taken together, these results indicated that ΔA146Ply induced apoptosis in KG-1 and C1498 cells by inhibiting autophagy.

### ΔA146Ply inhibits autophagy and induces apoptosis in a mouse model of AML

**Fig. 5:**
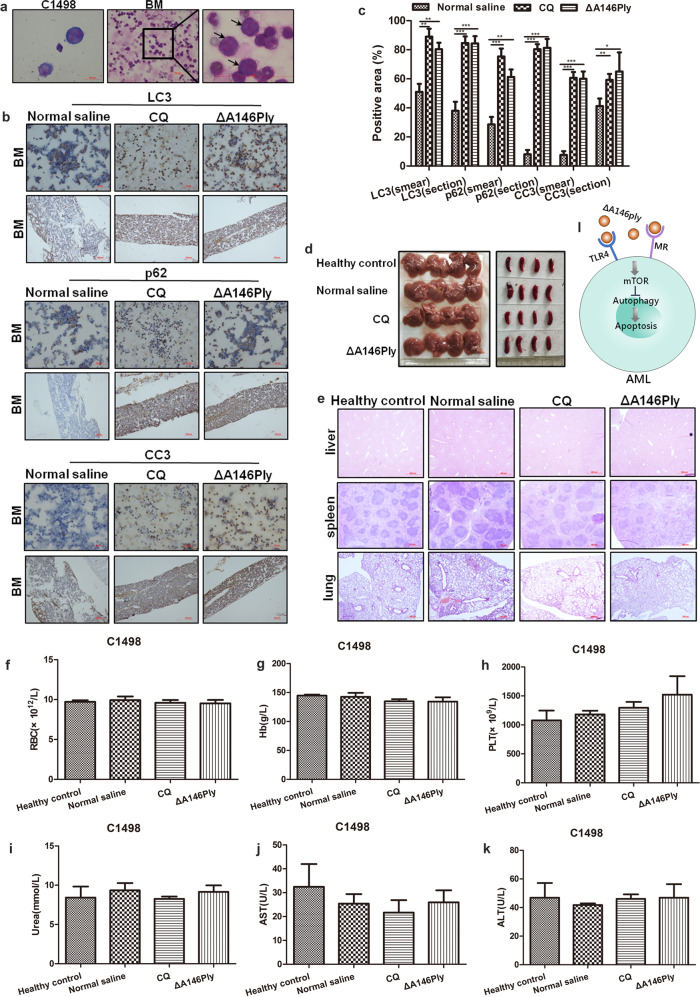
ΔA146Ply inhibits autophagy and induces apoptosis in a mouse model of AML. **a** Wright staining of cultured C1498 cells and bone marrow smears. **b** Immunohistochemical analysis of LC3, p62, and CC3 levels in bone marrow smears and sections. **c** The graph shows the mean percentage ± SD of the positive area (*n* = 3). **d** Pictures of liver and spleen tissues from euthanized mice. **e** Liver, spleen, and lung sections after HE staining. **f**–**h** Analysis of RBCs, hemoglobin, and PLTs. **i**–**k** Biochemical analysis of serum urea, AST, and ALT levels. **l** Schematic model demonstrating the effect of ΔA146Ply on AML cells. Student’s *t* test was used to analyze significant differences. *p < 0.05, **p < 0.01,***p < 0.001.

To determine whether ΔA146Ply can inhibit autophagy and induce apoptosis in vivo, a mouse model of AML was constructed by intravenous injection of C1498 cells into C57BL/6J mice, and CQ, the only autophagy inhibitor approved for in vivo use in humans to date, was used as a positive control. Twelve days after C1498 injection, the cells were detected in mouse bone marrow, demonstrating the successful establishment of the AML mouse model (Fig. 5a). Sixteen days after ΔA146Ply administration, femurs were collected from euthanized mice, and bone marrow smears and sections were prepared for immunohistochemical analysis. As shown in Fig. 5b, c, LC3, p62, and CC3 levels in the ΔA146Ply group were significantly higher than those in the medium group, and the effect was comparable to that of CQ, indicating that ΔA146Ply inhibited autophagy and induced apoptosis in a mouse model of AML.

To determine whether the mice could tolerate ΔA146Ply treatment, liver, spleen, and lung tissues and blood samples were collected for safety analysis. As shown in Fig. 5d, e, these tissues showed no obvious damage after CQ or ΔA146Ply administration. In addition, there was no significant difference in RBC counts, PLT counts, or hemoglobin levels among the healthy control, normal saline, CQ, and ΔA146Ply groups (Fig. 5f–h). Serum urea, AST, and ALT levels also did not differ among these groups (Fig. 5i–k). These results demonstrated that the mice tolerated ΔA146Ply treatment well. Figure 5l shows the working model of ΔA146Ply in AML cells.

### ΔA146Ply plus CQ synergistically inhibits autophagy and induces apoptosis

**Fig. 6:**
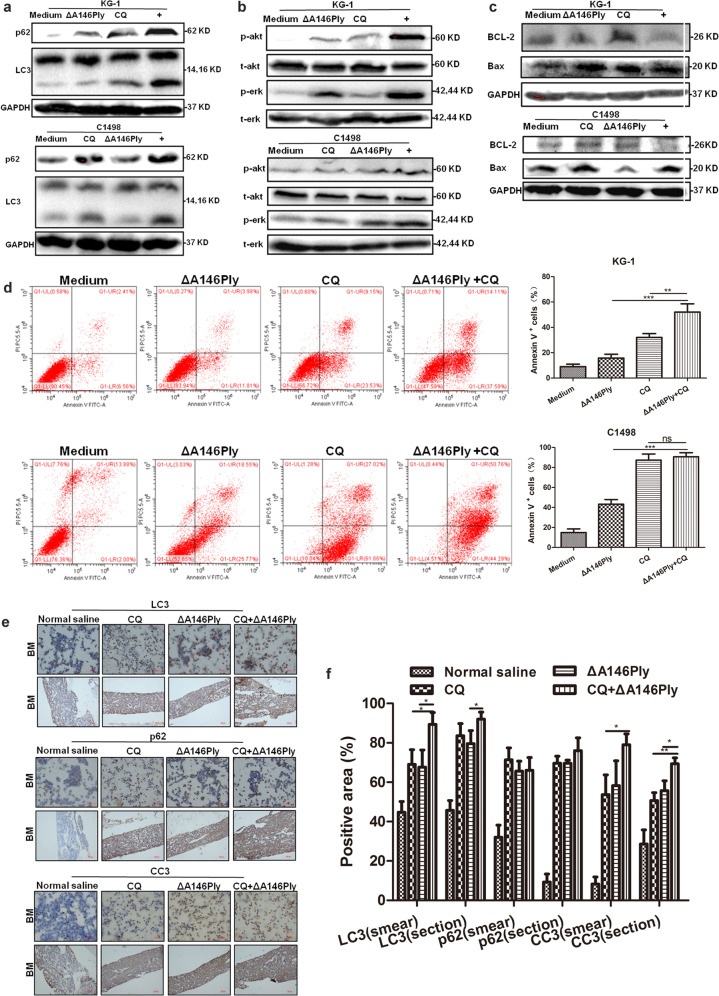
The combination of ΔA146Ply and CQ synergistically inhibits autophagy and induces apoptosis. **a**–**c** Intracellular proteins in KG-1 and C1498 cells treated with CQ (20 μM), ΔA146Ply (10 μg/ml), or their combination were measured by western blotting. **d** Flow cytometry was used to analyze apoptotic KG-1 and C1498 cells treated with CQ (20 μM), ΔA146Ply (10 μg/ml), or their combination for 48 h. Representative pictures are shown. The mean percentage ± SD of Annexin V^+^ cells is shown in the graphs (*n* = 3). **e** Immunohistochemical analysis of LC3, p62, and CC3 levels in bone marrow smears and sections. **f** The graph shows the mean percentage ± SD of the positive area (*n* = 3). Student’s *t* test was used to analyze significant differences. ns, no significance, *p < 0.05, **p < 0.01,***p < 0.001.

To determine whether the combination of ΔA146Ply and CQ could exert a synergistic effect on autophagy inhibition and apoptosis induction, KG-1 and C1498 cells were treated, and western blotting was performed to analyze intracellular autophagy- and apoptosis-associated proteins. The data in Fig. 6a show that p62 and LC3 levels in the combination group were higher than those in the single agent groups. The phosphorylation levels of Akt and ERK in KG-1 and C1498 cells were also higher in the combination group than in the single agent groups (Fig. 6b). After ΔA146Ply and CQ treatment, BCL-2 levels decreased and Bax levels increased compared with those in the medium group (Fig. 6c), indicating the initiation of the intrinsic apoptosis pathway. The percentage of apoptotic KG-1 cells was also higher in the combination group than in the single agent groups. After CQ treatment, the percentage of apoptotic C1498 cells was ~90%, suggesting hat there was no significant synergistic effect in the combination group (Fig. 6d). In a mouse model of AML, after ΔA146Ply and CQ treatment, LC3 and CC3 levels in the bone marrow were increased compared with those in the single agent groups (Fig. 6e, f).

**Fig. 7:**
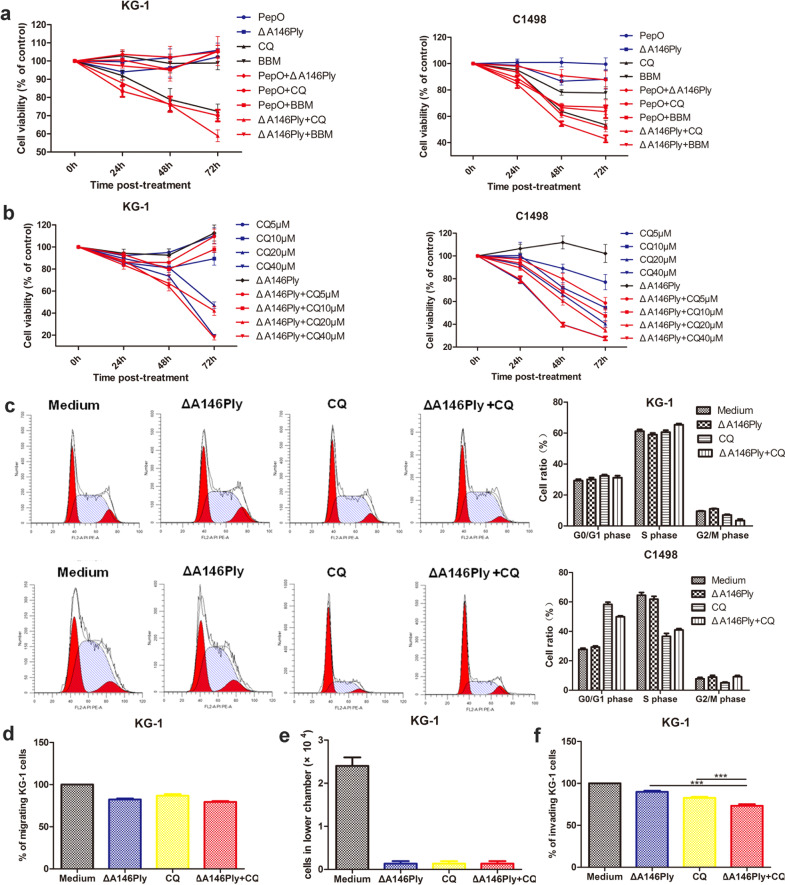
The effect of combination treatment on the proliferation, cell cycle, migration, and invasion of KG-1 and C1498 cells. **a**, **b** A CCK8 assay was performed to determine cell viability. The data are shown as the mean ± SD (*n* = 6). **c** Flow cytometry was used to analyze the distribution of the cell cycle in KG-1 and C1498 cells treated with CQ (20 μM), ΔA146Ply (10 μg/ml), or their combination for 48 h. Representative pictures are shown. The mean cell ratios ± SD at different phases are shown in the graphs (*n* = 3). After 24 h of treatment, the percentage of KG-1 cells migrating (**d**) or invading (**f**) through the Transwell insert is shown, and the control was set as 100% (*n* = 3). Cells that migrated through the Transwell insert and in the lower chamber were counted (**e**) (*n* = 3). Student’s t test was used to analyze significant differences. ***p < 0.001.

Whether the combination treatment affected the proliferation, cell cycle, migration, and invasion of KG-1 and C1498 cells was also examined. The data in Fig. 7a, b show that ΔA146Ply modestly enhanced CQ-mediated proliferation inhibition. PepO, a recombinant pneumococcal virulence protein, and berbamine (BBM), a new autophagy inhibitor, were used as controls. There was no significant synergistic effect on the cell cycle or migration in KG-1 and C1498 cells (Fig. 7c–e). However, the percentage of invading KG-1 cells was slightly lower in the combination group than in the single agent groups (Fig. 7f).

**Fig. 8:**
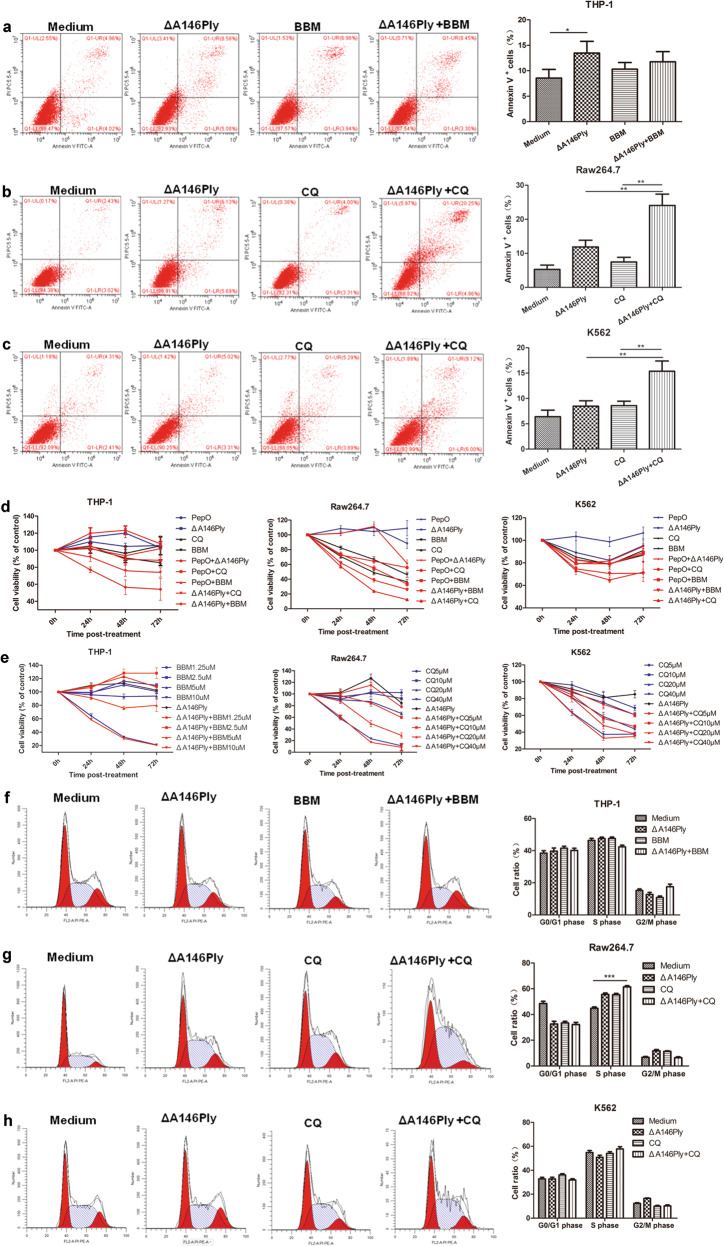
The effect of combination treatment on apoptosis, proliferation, and the cell cycle in THP-1, Raw264.7, and K562 cells. Flow cytometry was used to analyze apoptotic THP-1 (**a**), Raw264.7 (**b**), and K562 (**c**) cells. Representative pictures are shown. The mean percentage ± SD of Annexin V^+^ cells is shown in graphs (*n* = 3). **d**, **e** A CCK8 assay was performed to determine cell viability. The data are shown as the mean ± SD (*n* = 6). Flow cytometry was used to analyze the distribution of the cell cycle in THP-1 (**f**), Raw264.7 (**g**), and K562 cells (**h**) treated with ΔA146Ply (10 μg/ml), CQ/BBM (CQ, 20 μM; BBM, 5 μM), or their combination for 48 h. Representative pictures are shown. The mean cell ratios ± SD at different phases are shown in graphs (*n* = 3). Student’s *t* test was used to analyze significant differences. *p < 0.05, **p < 0.01, ***p < 0.001.

The effect of combination treatment on other leukemia cells, including THP-1, Raw264.7, and K562 cells, was also analyzed in the present study. The percentage of apoptotic THP-1 cells did not differ among the ΔA146Ply, CQ, and combination groups (Fig. 8a). However, the percentage of apoptotic Raw264.7 and K562 cells was higher in the combination group than in the single agent groups (Fig. 8b, c). ΔA146Ply enhanced the BBM-mediated proliferation inhibition at a dose of 5 μM and CQ-mediated proliferation inhibition at a dose of 20 μM in these cells (Fig. 8d, e). The combination treatment also did not show any synergistic effects on the cell cycle in these cells (Fig. 8f–h). These results indicated that ΔA146Ply and CQ synergistically inhibited autophagy and induced apoptosis.

## Discussion

In this study, we provide evidence that ΔA146Ply inhibits autophagy and induces apoptosis in KG-1 and C1498 cells and that it induces apoptosis by inhibiting autophagy. ΔA146Ply and CQ synergistically inhibited autophagy and induced apoptosis. Our study provides an alternative autophagy inhibitor that may be used for leukemia therapy.

Our previous study proved that ΔA146Ply inhibited autophagy in triple-negative breast cancer cells by activating TLR4 and MR. ΔA146Ply-mediated autophagy inhibition in AML cells expressing TLR4 and MR further suggests that ΔA146Ply is an effective autophagy inhibitor that targets TLR4 and MR. To date, CQ is the only autophagy inhibitor approved for use in humans^38^. In this study, we found that in a mouse model of AML, the ΔA146Ply-mediated autophagy inhibition was equal to that of CQ. CQ was administered every day, and ΔA146Ply was administered every 4 days. Therefore, we hypothesized that the effect of ΔA146Ply may be more durable. The safety of ΔA146Ply for in vivo use has been analyzed in different mouse tumor models. No obvious damage was found in the main internal organs, including the liver, kidney, spleen, and lung tissues. These results demonstrated that ΔA146Ply may be a safe and effective autophagy inhibitor with the potential to be a clinical agent.

Increasing evidence has demonstrated that microbiome-based therapy has unique advantages in treating malignant diseases^39–41^. In 1876, a mixture of *Pyogenic Streptococcus* and *Serratia marcescens* was injected to cure sarcoma by Wiliam B. Coley^42^. Bacillus Calmette-Guerin was approved for bladder cancer treatment in 1990^43^. Doxorubicin isolated from *Streptomyces peucetius* is used as a chemotherapeutic agent for multiple tumors^44^. Two main strategies, including intracellular signaling modulation and immunomodulation, are used by microorganisms and microbe-derived bioactive components to exert therapeutic effects^39,40^. The effect of ΔA146Ply, a microbe-derived bioactive component, on the modulation of intracellular signaling has been proven. Our previous study showed that among tumor-infiltrating lymphocytes, the percentage of CD8^+^ T cells significantly increased after ΔA146Ply administration in 4T1-bearing mice, suggesting its effect on immunomodulation^45^. However, the related mechanisms remain to be determined.

Previous studies have demonstrated that basal autophagy in leukemia cells determines their response to autophagy inhibition. Cells with low autophagy levels were more susceptible to HDACis, which inhibit autophagy. K562 cells with high autophagy levels were not susceptible to HDACi-induced apoptosis^28^. In our study, KG-1 and C1498 cells with low autophagy levels were more susceptible to ΔA146Ply treatment, and there was a significantly increased percentage of apoptotic cells. ΔA146Ply or CQ alone failed to induce apoptosis in K562 cells, which was consistent with previous studies. A possible reason for this failure may be that ΔA146Ply alone cannot inhibit autophagy to a level that is lower than the critical threshold, which leads to apoptosis induction. The combination of ΔA146Ply and CQ significantly induced apoptosis in K562 cells, which to some extent supports our hypothesis.

Interestingly, we found that the levels of LC3, p62, p-Akt, and p-ERK were higher in the combination group than in the single agent groups. The increases in LC3 and p62 levels indicated enhanced blockade of autophagic flux, and the increases in p-Akt and p-ERK levels indicated enhanced signal input for autophagic inhibition. However, the detailed mechanisms remain to be clarified. We also found that ΔA146Ply treatment inhibited the migration and invasion of KG-1 cells. Whether this effect resulted from autophagy inhibition remains to be determined.

In conclusion, our study provides evidence that ΔA146Ply may act as a novel autophagy inhibitor for leukemia therapy. The combination of ΔA146Ply and CQ, a clinically available autophagy inhibitor, synergistically inhibited autophagy.

## Data Availability

All data are presented in this paper.
